# Characterization of Outcomes by Surgical Management of Lung Neuroendocrine Tumors Associated With Cushing Syndrome

**DOI:** 10.1001/jamanetworkopen.2021.24739

**Published:** 2021-09-29

**Authors:** Kenneth P. Seastedt, George A. Alyateem, Karthik Pittala, Seth M. Steinberg, David S. Schrump, Lynnette K. Nieman, Chuong D. Hoang

**Affiliations:** 1 Department of Surgery UHS F. Edward Hébert School of Medicine, Bethesda, Maryland; 2 Now with Department of Surgery, Beth Israel Deaconess Medical Center, Boston, Massachusetts; 3 Thoracic Surgery Branch, National Cancer Institute, Bethesda, Maryland; 4 Biostatistics and Data Management Section, Center for Cancer Research, National Cancer Institute, National Institutes of Health, Bethesda, Maryland; 5 Diabetes, Endocrine and Obesity Branch, National Institute of Diabetes and Digestive and Kidney Diseases, Bethesda, Maryland

## Abstract

**Importance:**

Ectopic adrenocorticotropic hormone secretion from lung tumors causing Cushing syndrome are associated with high rates of morbidity. Optimal management remains obscure because knowledge is based on rare reports with few patients.

**Objective:**

To characterize the outcomes of lung neuroendocrine tumors associated with Cushing syndrome.

**Design, Setting, and Participants:**

An observational case series review from 1982 to 2020 was conducted in a single institution referral center. Kaplan-Meier analysis estimated disease-free survival (DFS). Participants underwent curative-intent surgery for a lung neuroendocrine tumor causing Cushing syndrome.

**Exposures:**

Lobectomy or pneumonectomy vs sublobar resection.

**Main Outcomes and Measures:**

Disease-free survival, disease persistence/recurrence.

**Results:**

Of the 68 patients, the median age was 41 years (range, 17-80 years), 42.6% (29 of 68) were male, 81.8% (54 of 66) were White, with a mean follow-up after surgery was 16 months (range, 0.1-341 months). Lobectomy was the most common procedure (48 of 68 [70.6%]), followed by wedge resection (16 of 68 [23.5%]) and segmentectomy (3 of 68 [4.4%]). Video-assisted thoracoscopic surgery was performed in 19 of 68 (27.9%) of patients. Surgical morbidity was 19.1% (13 of 68), and perioperative mortality was 1.5% (1 of 68). Lymph node positivity was 37% (22 of 59) when evaluable. The overall incidence of persistence/recurrence was 16.2% (11 of 68) with a median time to recurrence of 55 months (range, 18-152 months). The median DFS was reached in 12.7 years (0.1-334 months). There were no statistical differences in DFS based on tumor size, stage (8th edition TNM), whether full systematic lymphadenectomy was performed or not, nodal status, or surgical approach.

**Conclusions and Relevance:**

In this case series study, neuroendocrine pulmonary tumors associated with Cushing syndrome had increased nodal metastasis, higher recurrence, and lower DFS than quiescent bronchopulmonary carcinoid tumors, but many patients experienced favorable outcomes. This observation is underscored by the discordance of TNM-stage classifications vs prognosis. Observing no difference in surgical techniques, the implication may be that a lung-sparing approach could suffice. These results may reflect the intrinsic importance of the hormone physiology instead of the carcinoid biologic factors.

## Key Points

**Question:**

What is the optimal surgical management of ectopic adrenocorticotropic hormone secreting pulmonary carcinoid tumors associated with Cushing syndrome (CS)?

**Findings:**

In this case series study of 68 patients with Cushing syndrome, ectopic adrenocorticotropic hormone–secreting carcinoid tumors with CS were associated with increased metastasis to lymph nodes, higher recurrence, and lower overall disease-free survival at 5 and 10years compared with quiescent bronchial carcinoid tumors, irrespective of histologic subtype, with no difference in outcome based on surgical approach.

**Meaning:**

Results of this study suggest that underlying carcinoid biologic factors may not be as important as the symptomatic hormonal physiology, and a lung-sparing surgical approach coupled with routine lymphadenectomy may be an optimal intervention when CS resolves.

## Teaser

This case series study compares survival and disease recurrence of patients with lung neuroendocrine tumors associated with Cushing syndrome treated using lobectomy or pneumonectomy vs sublobar resection.

## Introduction

Pulmonary neuroendocrine tumors, or bronchopulmonary carcinoid tumors, account for 1% to 2% of lung cancers in the US. Approximately 90% are typical carcinoid (TC) tumors, and 10% are atypical carcinoid (AC) tumors.^1,2^ Given the neuroendocrine origin of these tumors, various paraneoplastic syndromes may occur. Cushing syndrome (CS) from ectopic adrenocorticotropic hormone secretion (EAS) is seen in 1% to 6% of carcinoid tumors worldwide, comprising a rare subgroup.^3^

Surgical resection is the standard treatment and is curative in selected patients with CS with EAS bronchial carcinoid tumors.^4,5^ However, there are no uniform surgery guidelines, which vary from wedge resection to pneumonectomy to bronchial sleeve resection for central tumors.^6^

Mediastinal lymph node sampling or dissection is controversial in lung carcinoid management. Nodal dissection has not been strictly adhered to, given the low metastatic potential of hormone-quiescent TC and AC.^7^ Thoracic carcinoid tumors producing CS have been reported in a few series to have higher rates of nodal involvement,^8^ with subsequent prevailing concerns these represent an aggressive variant of carcinoid tumor and require more radical surgical management.^9^ However, their aggressiveness has been questioned,^10^ obscuring prognosis.^11^

To better characterize the outcomes, we assessed 68 patients whose EAS bronchial carcinoid tumors caused CS. The aim of this case series study is to describe our current diagnostic approach and evaluate the utility of various therapeutic strategies.

## Methods

We reviewed demographic and clinical data from 68 patients with CS with EAS from a thoracic lesion as evaluated by National Institutes of Health endocrinologists, who underwent curative-intent surgery between 1982 to 2020 by thoracic surgeons (National Cancer Institute). Missing data were ignored, noted where applicable, and not replaced. All patients provided informed consent for participation in protocols approved by the National Institutes of Health. This case series study follows the reporting guideline for case series through clear hypothesis investigation, delineation of eligibility criteria, description of treatments, comparison with external cohorts, statistical analysis and assumptions where appropriate, discussion of new hypotheses given the results, and discussion of the limitations and future directions.^12^

The approach at the National Institutes of Health to confirm CS consisted of routine laboratory evaluation according to the Endocrine Society Clinical Practice Guidelines^13^ (eMethods and eFigure in the Supplement). The surgeon decided the type of procedure, the extent of lung resection, and retrieval of lymph nodes. Bronchoscopy assessed for endobronchial involvement.

Patient characteristics were compared between those with a lobectomy vs other procedures using a Fisher exact test for 2 groups, the Mehta modification to Fisher exact test for more than 2 unordered categories, an exact Cochran-Armitage test for trend to compare ordered categories, and a Wilcoxon rank sum test when comparing continuous variables.

The Kaplan-Meier method was used to estimate the probability of disease-free survival (DFS) as a function of time. Log-rank tests were used to determine the degree of statistical difference between pairs of curves or to provide a global *P* value for jointly evaluating a set of curves. All analyses were performed using SAS version 9.4 (SAS Institute Inc.). Median values are presented for continuous variables given the small sample size and nonnormal distributions. Overall survival was not reportable in this study because this could not be evaluated meaningfully, with many patients last known to be alive more than 10 years ago.

## Results

### Baseline Characteristics

eTable 1 in the Supplement summarizes the demographic variables and preoperative evaluations of our cohort. Additional data are available in the eResults in the Supplement. Of the 68 patients, the median age was 41 years (range, 17-80 years) (compared with a median age of 60 years in non-EAS carcinoid tumors^13^), 42.6% (29 of 68) were male, 81.8% (54 of 66) were White, with a median follow-up after surgery of 16 months (range, 0.1-341 months). There was an absence of obstructive bronchial symptoms more typical of pulmonary carcinoid tumors without endocrinopathy. eTable 2 in the Supplement lists the presenting signs and symptoms of CS in the cohort.

### Surgical Management

Surgical treatment and tumor characteristics are presented in Table 1. The median time from diagnosis to surgery was 2 months (range, 1-144 months), and the median length of stay after surgery was 10 days (range, 4-52 days). Lobectomy was the most common procedure (48 of 68 [70.6%]), followed by wedge resection (16 of 68 [23.5%]), and segmentectomy (3 of 68 [4.4%]). Video-assisted thoracoscopic surgery was performed in 19 of 68 (27.9%) patients, of which 15 were performed since 2015. Nodal staging was performed at the time of surgery in 59 of 68 patients (86.8%), with 1 surgeon accounting for 6 of 9 of patients without any nodal staging from 2009 to 2011. Of these patients without nodal staging, 6 of 9 were T1a, 2 of 9 T1b, 1 of 9 AC, and 1 case of disease persistence (CS) after tumor removal. Of the 59 patients with intraoperative nodal assessment, there were 6 patients with partial nodal dissection of lobar nodes (N1) who did not undergo systematic lymph node mapping. In this subgroup of 6 patients, 2 had tumor recurrence, both occurring in the ipsilateral hilum. In total, 15 of 68 of patients (22.1%) were incompletely staged with less than a systematic lymph node assessment.

**Table 1.** Surgical Treatment and Tumor Characteristics

Characteristic	No./total No. (%)
Surgical approach by tumor subtype	
Typical carcinoid	57/68 (83.8)
Lobectomy	40/57 (70.2)
Wedge	13/57 (22.8)
Segmentectomy	3/57 (5.3)
Pneumonectomy	1/57 (1.7)
Atypical carcinoid	11/68 (16.2)
Lobectomy	8/11 (72.7)
Wedge	3/11 (27.3)
T Status	
1a	38/68 (55.9)
1b	28/68 (41.2)
1c	1/68 (1.5)
2a	1/68 (1.5)
Pathologic stage^a^	
IA1	22/59 (37.3)
IA2	14/59 (23.7)
IA3	1/59 (1.7)
IIB	10/59 (16.9)
IIIA	12/59 (20.3)
Unknown (NNS)	9/68 (13.2)
Tumor subtype and nodal status	
Typical carcinoid	57/68 (83.8)
N0	34/49 (69.4)
N1	9/49 (18.4)
N2	6/49 (12.2)
NX	8/57 (14.0)
Atypical carcinoid	11/68 (16.2)
N0	3/10 (30.0)
N1	1/10 (10.0)
N2	6/10 (60.0)
NX	1/11 (9.1)

Abbreviation: NNS, nodes not sampled.

^a^ Six patients had partial lymphadenectomy (N1 only), and stage based on partial nodal staging.

### Outcomes

Overall morbidity following surgery was 13 of 68 (19.1%). Complications included 4 patients with atrial fibrillation, 1 patient with persistent air-leak requiring chemical pleurodesis, 1 with adrenal insufficiency, 1 with pneumothorax requiring replacement of a chest tube, 1 with loculated effusion requiring pigtail tube drainage, and 1 with upper gastrointestinal bleed. Two chyle leaks occurred, and 1 patient required thoracic duct ligation. One patient had a prolonged intensive care unit stay owing to respiratory failure and severe steroid myopathy. One patient died of cardiac arrest on postoperative day 8, so overall mortality was 1 of 68 (1.5%).

To evaluate the potential association between the extent of surgical resection and outcomes, Table 2 compares patients undergoing lobectomy or pneumonectomy (49 of 68 [72.1%]) vs wedge resection or segmentectomy (19 of 68 [27.9%]). There was no difference in the rate of persistence/recurrence of disease (as a combined outcome) between the categories, with the sublobar cohort significantly older than the lobectomy cohort (sublobar cohort median age, 55 years [range, 17-80 years] vs lobectomy cohort median age, 36 years [range, 19-70]; *P* = .01). There was a pattern of statistically more node-positive AC tumors in the lobectomy group than in the other patients (7 vs 0 overall; *P* = .01). eTable 3 in the Supplement lists the characteristics of these sublobar patients.

**Table 2.** Comparison of Patients Undergoing Lobectomy With Patients Undergoing Wedge Resection or Segmentectomy

Variable	No./total No. (%)		*P* value
	Lobectomy^a^	Wedge resection or segmentectomy	
Patients	49/68 (72.1)	19/68 (27.9)	NA
Age, median (range), y	36 (19-70)	55 (17-80)	.01
Sex			
Male	22/49 (44.9)	7/19 (36.8)	.60
Female	27/49 (55.1)	12/19 (63.2)	
FEV_1_ (%): median (range)	87 (54-116)^b^	90 (31-116)^c^	.74
Race			
White	37/49 (75.5)	17/19 (89.5)	.64
Black	6/49 (12.2)	2/19 (10.5)	
Hispanic	4/49 (8.2)	0/19	
Unknown	2/49 (4.1)	0/19	
Pathologic stage^d^			
IA1	17/48 (35.4)	5/11 (45.4)	.36
IA2	11/48 (22.9)	3/11 (27.3)	
IA3	1/48 (2.1)	0/11	
IIB	8/48 (16.7)	2/11 (18.2)	
IIIA	11/48 (22.9)	1/11 (9.1)	
Unknown (NNS)	1/49 (2.0)	8/19 (42.1)	
Typical carcinoid	41/49 (83.7)	16/19 (84.2)	>.99
N0	29/41 (70.7)	5/8 (62.5)	.79^e^
N1	7/41 (17.1)	2/8 (25.0)	
N2	5/41 (12.2)	1/8 (12.5)	
NNS	0/41	8/16 (50.0)	
Atypical carcinoid	8/49 (16.3)	3/19 (15.8)	>.99
N0	0/7	3/3 (100)	.01^e^
N1	1/7 (14.3)	0/3	
N2	6/7 (85.7)	0/3	
NX	1/8 (12.5)	0/3	
Persistence/recurrence	8/49 (16.3)	3/19 (15.8)	>.99^f^
Recurrence	6/49 (12.2)	1/19 (5.3)	
Persistence	2/49 (4.1)	2/19 (10.5)	
Follow-up, median (range)	19 (1-341)	13 (1-143)	.09

Abbreviations: FEV_1_, forced expiratory volume in 1 second; NA, not applicable; NNS, nodes not sampled.

^a^ Lobectomy group includes 1 pneumonectomy.

^b^ n = 12.

^c^ n = 13.

^d^ Six patients had partial lymphadenectomy (N1 only), and stage based on partial nodal staging.

^e^ *P* value for comparison of increasing N0-N2 values between the 2 surgical groups.

^f^ Comparison of persistence/recurrence outcome considered as a binary variable at last follow-up; may not reflect ultimate recurrence distribution if additional follow-up was to take place.

The 5-year DFS for patients who underwent operative intervention with curative intent was 73.4% (95% CI, 48.7%-87.6%), and 10-year DFS was 55.1% (95% CI, 26.3%-76.5%). Stratified by histology, the 5-year DFS for TC was 75.4% (95% CI, 49.2%-89.3%) and the 10-year DFS for TC was 50.2% (95% CI, 18.3%-75.7%), and for AC the 5-year DFS was 75.0% (95% CI, 12.8%-96.0%) and the 10-year DFS was 75.0% (95% CI, 12.8%-96.0%). These differences of DFS among histologic subtypes were not significant. The median DFS was reached 13 years from the date of surgical treatment. Furthermore, there were no statistical differences in DFS based on tumor size, stage, whether full systematic lymphadenectomy was performed or not, nodal status, or surgical approach (Figure).

**Figure.**
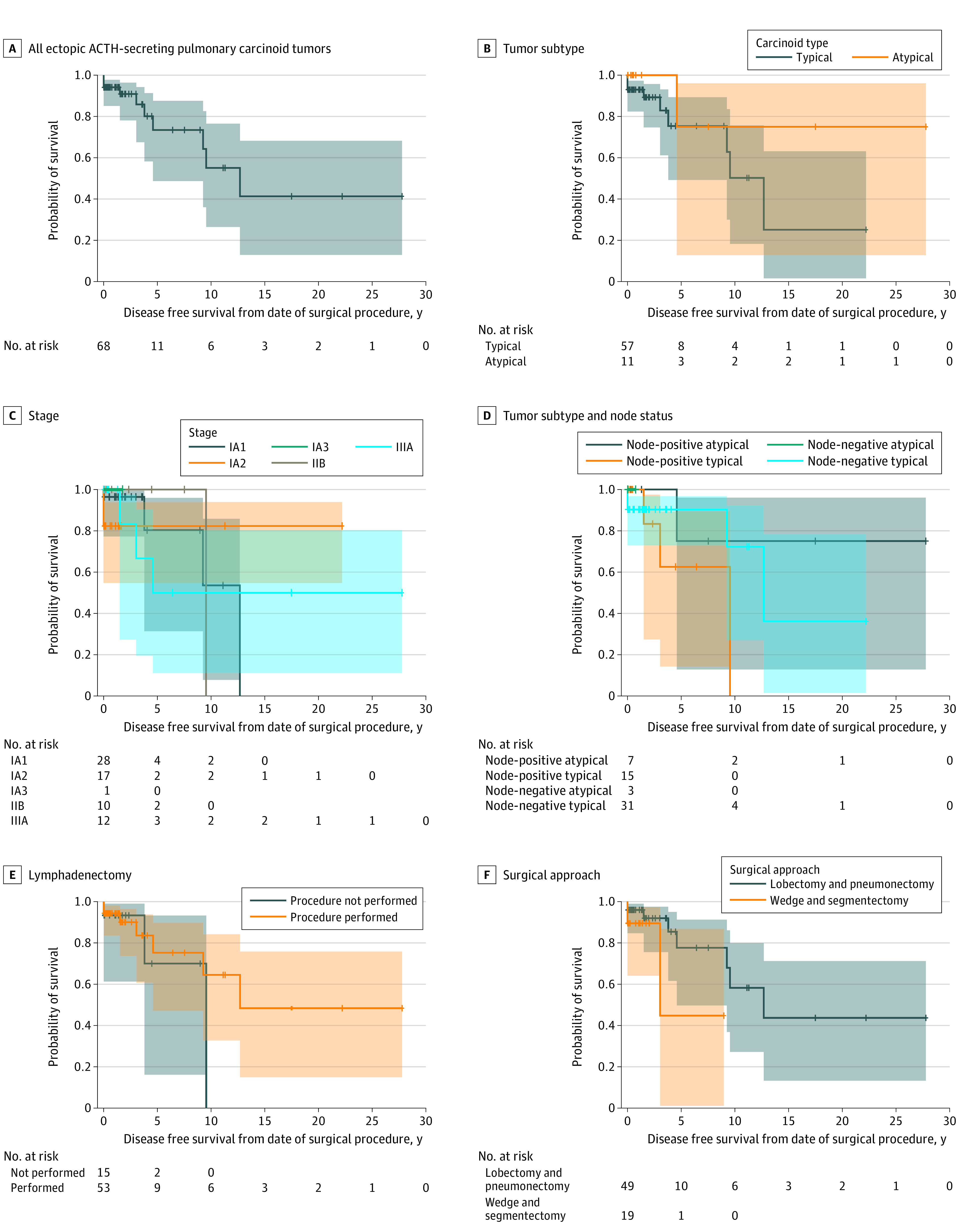
Time to Recurrence in Ectopic Adrenocorticotropic Hormone-Secreting Bronchial Carcinoid Tumors

Median follow-up after surgery was 16 months (range, 0.1-341 months). At the time of the last follow-up, 52 of 68 patients (76.4%) were alive and tumor free, 2 of 7 were alive and tumor free after treatment of persistent/recurrent tumor, 7 of 68 were alive with persistent/recurrent disease, 7 of 68 were dead and tumor free, and 2 of 68 were dead with persistent disease.

### TNM Staging of Lung Tumors With Cushing Syndrome

The TNM staging criteria was not associated with clinical outcome. T1a (n = 38) vs T1b (n = 28) could not be differentiated by DFS probabilities. Neither mediastinal nor thoracic lymph node involvement were associated with DFS when analyzed by N1 vs N2 vs any N positivity. Most patients had systematic lymph node assessment during surgical resection, yet DFS results indicate this did not statistically matter in terms of outcomes. In contrast, the pathologic lymph node status is well known to predict mortality in quiescent bronchial carcinoid tumors.^14^ Also, unlike quiescent bronchial carcinoid tumors, histology did not impact our cohort with no statistical difference in DFS probabilities among TC vs AC. When DFS was assessed according to N status and histology, there were no statistical differences among any combinations of N and TC or AC. In our series, no single particular surgical approach was superior compared with others. Furthermore, neither the surgical approach (minimally invasive or open) nor extent of lung resection (lobectomy or sublobar) was associated with any major difference in outcomes. Lastly, we found no significant differences in DFS regarding age, sex, race, pTNM stage, grade, or time to surgery.

### Persistent/Recurrent Disease Patterns and Treatments

The overall incidence of persistence/recurrence in this case series was 11 of 68 (16.2%), with features of these patients listed in eTable 4 and eTable 5 in the Supplement. Of these patients, 4 had persistent disease (R0 resection, but with CS postoperatively), 7 were recurrent disease (R0 resection without CS postoperatively), 10 of 11 (90.9%) were TC, and 1 of 11 (9.1%) were AC. The development of recurrent disease occurred in 6 of 57 (10.5%) of all patients with TC, 1 of 11 (9.1%) of all patients with AC, and 4 of 22 (18.2%) of all patients with node-positive disease. For the 7 patients who were noted to recur, the mean time to recurrence was 76 months (>6 years) with a median of 55 months (range, 18-152 months) (n = 7).

Median follow-up for patients with recurrent disease after surgery was 175 months (range, 87-341 months). At the time of the last follow-up, 1 of 7 were alive and tumor-free, 5 of 7 were alive with disease, and 1 of 7 were dead with persistent disease. Median follow-up for patients with persistent disease after surgery was 14 months (range, 1-117 months) and, at the time of last follow-up, 1 of 4 were alive and tumor free, 1 of 4 were alive with disease, and 2 of 4 were dead with persistent disease.

### Comparative Analysis of Series

To place the National Institutes of Health results in context, we reviewed pooled results from other surgical series (>5 patients in each) and present the findings in Table 3 and Table 4.^9,10,11^ The findings in Table 4 indicate that most tumor traits did not vary substantially across the surgical series. In summary, from Table 3 and Table 4, primarily young patients are affected (median age, 39 years), surgical morbidity is relatively low at 21 of 112 (19%), perioperative mortality is rare at 1 of 112 (0.9%), and lifetime follow-up is feasible. TC are more commonly associated with EAS causing CS in 94 of 112 (84%) cases, tumors are typically small (mean diameter about 1.0 cm), and neuroendocrine metastasis to lymph nodes is markedly high, affecting 39 of 99 (39%). The rate of persistence/recurrence is modest, affecting approximately 20 of 111 patients (18%).

**Table 3.** Comparison Between Adrenocorticotropic Hormone–Secreting Carcinoid Series

Characteristics	No./total No. (%)				
	NIH 2021	HEGP 2011	Mayo 2005	MGH 1997	Total
Patients	68	14	23	7	112
Age, mean (range), y	43 (17-80)	40 (16-63)	39 (14-71)	39 (20-74)	
Male	29/68 (42.6)	8/14 (57.1)	10/23 (43.5)	5/7 (71.4)	52/112 (46.4)
Tobacco use	9/30 (30.0)	7/14 (50.0)	10/23 (43.5)	NR	26/67 (38.8)
Bronchial symptoms	0/68 (0)	1/14 (7.1)	4/23 (17.4)	0/7 (0)	5/112 (4.5)
Carcinoid syndrome	0/68 (0)	0/14 (0)	NR	1/7 (14.3)	1/89 (1.1)
Abnormal pituitary MRI	12/65 (18.5)	4/14 (29.0)	NR	1/5 (20)	17/84 (20.2)
Petrosal sinus catherization gradient	0/14 (0)	0/6 (0)	NR	1/5 (20)	1/25 (4.0)
Abnormal chest x-ray result	7/41 (17.1)	2/14 (14.0)	13/23 (56.5)	1/7 (14.3)	23/85 (27.1)
Bronchoscopic detection	2/19 (10.5)	3/10 (30.0)	1/14 (7.1)	0/7 (0)	6/50 (12.0)
Chest CT sensitivity	62/68 (91.2)	9/14 (64.3)	20/20 (100)	7/7 (100)	98/109 (89.9)
Octreotide scintigraphy	25/42 (59.5)	7/12 (58.3)	1/2 (50.0)	2/2 (100)	35/58 (60.3)
Adrenalectomy	11/68 (16.2)	3/14 (21.4)	7/23 (30.4)	1/7 (14.3)	22/112 (19.6)
Hypophysectomy	8/68 (11.8)	1/14 (7.1)	7/23 (30.4)	3/7 (42.9)	19/112 (17.0)
Duration before operation, mean (range), mo	13 (1-144)	33 (3-136)	17 (1-228)	24 (6-84)	
Lobectomy/pneumonectomy	48/68 (70.6)	12/14 (85.7)	18/23 (78.3)	3/7 (42.9)	81/112 (72.3)
Segmentectomy	3/68 (4.4)	1/14 (7.1)	4/23 (17.4)	1/7 (14.3)	9/112 (8.0)
Wedge resection	16/68 (23.5)	1/14 (7.1)	1/23 (4.3)	3/7 (42.9)	21/112 (18.7)
Lymphadenectomy	59/68 (86.8)^a^	14/14 (100)	19/23 (82.6)	4/7 (57.1)	96/112 (85.7)
Morbidity	13/68 (19.1)	2/14 (14.3)	6/23 (26.1)	0/7 (0)	21/112 (18.7)
Mortality	1/68 (1.5)	0/14 (0)	0/23 (0)	0/7 (0)	1/112 (0.9)
Length of stay (range), d	10 (4-52)	10 (6-22)	8 (4-28)	NR	
Follow-up, mean (range), mo	53 (1-341)	59 (3-174)	78^b^ (1-432)	59 (9-180)^c^	

Abbreviations: HEGP, Hôpital Européen Georges-Pompidou; Mayo, Mayo Clinic; MGH, Massachusetts General Hospital; NIH, National Institutes of Health; NR, not reported.

^a^ 6 patients had partial lymphadenectomy (N1 only).

^b^ Median.

^c^ Follow-up for 6 of 7 patients reported.

**Table 4.** Adrenocorticotropic Hormone–Secreting Carcinoid Series Subtype, Size, Nodal Status, and Recurrence

Institution	No.	Typical, No./total No. (%)	Mean size (range), cm	No./total No. (%)				
				N1	N2	N+	Recurrence	Persistence
MGH (1997)	7	5/7 (71.4)	1.5^a^ (0.7-2.5)	1/7 (14.3)	3/7 (42.9)	4/7 (57.1)	2/7 (28.6)	2/7 (28.6)
Mayo (2005)	23	21/23 (91.3)	1.3^b^ (0.3-10)	4/19 (21.0)	2/19 (10.5)	6/19 (31.6)	5/22 (22.7)	0/22 (0)
HEGP (2012)	14	11/14 (78.6)	0.4 (0.5-3.0)	3/14 (21.4)	4/14 (28.6)	7/14 (50.0)	0/14 (0)	0/14 (0)
NIH (2021)	68	57/68 (83.8)	1.1 (0.1-3.5)	10/59 (16.9)	12/59 (20.3)	22/59 (37.3)^c^	7/68 (10.3)	4/68 (5.9)
Total	112	94/112 (83.9)	1.1 (0.1-10)	18/99 (18.2)	21/99 (21.2)	39/99 (39.4)	14/111 (12.6)	6/111 (5.4)

Abbreviations: HEGP, Hôpital Européen Georges-Pompidou (France); Mayo, Mayo Clinic (Rochester); MGH, Massachusetts General Hospital; NIH, National Institutes of Health (current series); NR, not reported.

^a^ Based on preoperative imaging.

^b^ Median size.

^c^ Six patients had partial lymphadenectomy (N1 only).

Our results suggest that TC is the predominant histologic finding (5:1 vs AC) involved with EAS manifesting as CS in patients of mid-40s age, approximately 22 of 59 (37%) of cases have lymph node positivity for metastasizing neuroendocrine tumor (31% of TC and 70% of AC), and disease persistence/recurrence was less than 20% overall (91% TC and 9% AC). Of the recurrent tumors, 6 of 7 (85.7%) were TC. The time to recurrence was prolonged, taking more than 6 years on average. When disease recurred because of a metachronous tumor, this usually manifested as locoregional involvement in 6 of 7 (85.7%). The DFS at 5 and 10 years for TC was 75% and 50%, respectively, while for AC the 5- and 10-year DFS was 75% and 75%, respectively. Thus overall, the 5-year DFS for patients who underwent operative intervention with curative intent was 73%, and 10-year DFS was 55%.

## Discussion

This review of 68 patients with EAS lung carcinoid tumors and CS aimed to recharacterize these tumors to gain insights on treatment and outcomes in this rare subgroup. To date, this is the largest series from a single institution accompanied by long-term follow-up data (median follow-up after surgery, 16 months [range, 0.1-341 months], postsurgery and >10 years, persistent/recurrent disease). In contrast, the next largest series had 23 cases with mean follow-up of 6.5 years.^10^

For context, we reviewed the data from resected bronchial carcinoid tumors without CS. An analysis from the National Cancer Database (n = 3335) reported overall nodal involvement of 21% (17% of TC and 46% of AC).^14^ Generally, tumor recurrence occurs in about 10% of cases (3% of TC and 25% of AC).^14,15^ The 5- and 10-year DFS probabilities for TC were 92% and 85%, respectively, vs for AC, DFS probabilities were 72% and 32%, respectively (overall DFS 5- and 10-year 88% and 72%, respectively).^16^ Comparing these outcomes to our series, EAS carcinoid tumors with CS show increased nodal positivity across both histologic subtypes of lung neuroendocrine tumors, show an increased incidence of recurrence where, conversely, TC predominate over AC, and lastly, show worse 5- and 10-year overall DFS probabilities. Given the small number of AC in our series, the observation of similar 5- and 10-year DFS (75%) for AC should be interpreted with caution.

These contrasting clinicopathologic characteristics of EAS carcinoid tumors with CS compared with hormone-quiescent bronchial carcinoid tumors have been observed in the few other series.^9,10,11^ The consensus was that EAS carcinoid tumors represent a distinct, more aggressive subtype of lung tumor based on nodal involvement and recurrence. They advocated for an intensive surgical approach to include anatomic resection and radical lymphadenectomy. However, none of them reconciled the clinical paradox that EAS carcinoid tumors have a high cure rate with prolonged survival despite positive lymph nodes. Invoking the notion of more aggressive biology (vaguely defined from the outset^9^) may not be accurate or clinically applicable for these EAS carcinoid tumors, which do not recur for years (ie, >6 years in our series). By contrast, the median time to recurrence in nonhormonally active lung carcinoid tumors is less than 2 years.^17^ From our long-term follow-up of patients with EAS with persistence/recurrence, 8 of 11 (73%) remain alive.

These results suggest that testable hypotheses (related to staging, tumor hormone sequelae, and extent of lung resection) may help better characterize EAS lung carcinoid tumors and help establish quantitative parameters for improving outcomes.

Foremost is that the current AJCC staging is not well-suited to classify these hormonally active lung tumors. Given the lack of any statistical associations in DFS analysis stratified by multiple criteria (T, N, stage, and grade), it can be supposed that these tumors require a different set of parameters to align prognostically within AJCC. There is no accounting for adrenocorticotropic hormone levels, which directly reflect the hormonal physiologic factors producing the morbid adverse effects in this subgroup. Similarly, there is no accounting in the current AJCC schema of the tumor proliferation index Ki-67. Thus, in addition to identifying more meaningful staging variables reflecting the tumor biology in EAS lung carcinoid tumors, there is a need for variables that correlate with the unique hormone physiology manifested.

In contrast with conventional surgical notions for lung tumors regardless of indolent (eg, carcinoid tumors) or nonindolent (eg, non–small cell lung carcinoma) biologic factors is the prospect of not requiring formal anatomic resection, which, over the long-term, risks removing too much parenchyma in a cohort of younger patients. Our results, unconstrained by histologic considerations, show no difference in DFS outcomes that depend on surgical approach, the extent of lung resected (assuming complete negative margins), and whether lymph nodes were assessed intraoperatively. Because carcinoid tumors are small (detectable earlier because of endocrinopathy), a refocus on achieving negative margins with the simultaneous goal of maximal lung-sparing resection may represent a highly effective therapy with minimal morbidity. This surgical objective is better aligned with current surgical concepts in which there is a rationale to pursue sublobar resection for small tumors,^18^ although definitive results of the phase III trial (CALGB 140503) are not yet available. The DFS result of whether lymph nodes were assessed during lung resection should be interpreted with caution because the patient numbers in this subanalysis were very low. Although to our knowledge little evidence supports routine radical nodal dissection in quiescent TC tumors,^19^ in all EAS carcinoid cases there is cause to perform a systematic lymphadenectomy because of the intrinsic higher rate of node positivity. In the context of this regional metastatic phenomenon, it is especially important to minimize residual EAS foci in the thorax by routinely removing locoregional lymph nodes.

### Limitations

This study has limitations. Foremost, with this rare cancer, the number of cases remained low and, thus, was a limiting factor in all statistical calculations as evidenced by wide CIs and should be evaluated with caution. Of note, there were only 11 patients with AC in which 1 case had tumor recurrence, and there were small numbers of patients with node-positive disease. Some of our reported percentages (and denominator values) may be discordant with true occurrences in the general population. Additionally, many of the paper records were from decades ago, and some medical records were missing data. The National Institutes of Health referral nature led to some patients getting treatment here but then continuing care at their home institution. Therefore, some follow-up periods were truncated and this may contribute to the miscataloging of disease recurrences. Also, there is heterogeneity in surgical techniques of different surgeons over a long period. In addition, the retrospective nature of this study may be affected by patient selection bias and time-trend bias. However, given the rarity of these tumors, an inherent challenge remains in conducting prospective randomized clinical trials.

## Conclusions

In this case series, EAS carcinoid tumors with CS appear to be associated with increased metastasis to lymph nodes, higher recurrence (mostly local), and lower overall DFS at 5 and 10 years than quiescent bronchial carcinoid tumors, irrespective of histologic subtype. Nevertheless, we contend these tumors are not biologically aggressive since these patients have distinct, prolonged survival and delayed time to recurrence. Interestingly, we observed no difference in DFS based on surgical resection techniques. Also, we noted that the current staging system applied to these tumors raises questions about prognostic accuracy. Extrapolation may suggest that the underlying carcinoid biology may not be as important as the symptomatic hormonal physiology. Future studies may test whether a lung-sparing surgical approach coupled with routine lymphadenectomy is an optimal intervention in this scenario when normal endocrine functioning is restored and CS sequelae resolve.

## Notes

Supplement. **eMethods.** Protocol Approval, Surgical Techniques, Tumor Review, and Analysis **eResults.** Baseline Characteristics, Imaging Studies, Pathology, and Persistent/Recurrent Disease Patterns and Treatments
eReferences
**eTable1.** Demographic Variables and Pre-operative Evaluations **eTable2.** Pulmonary Neuroendocrine ACTH-Secreting Tumor Clinical Signs/Symptoms **eTable3.** Wedge/ Segmentectomy Only Patient Characteristics **eTable4.** Characteristics of Patients With Recurrent Disease **eFigure.** Approach to the Diagnosis of Cushing’s Syndrome Secondary to Ectopic ACTH Secretion
