# Supplementary material for: Characterization of Outcomes by Surgical Management of Lung Neuroendocrine Tumors Associated With Cushing Syndrome
Source: JAMA Netw Open. 2021 Sep 29;4(9):e2124739. doi: 10.1001/jamanetworkopen.2021.24739 (PMC8482056; doi:10.1001/jamanetworkopen.2021.24739)
Supplement: Supplement. — eMethods. Protocol Approval, Surgical Techniques, Tumor Review, and Analysis eResults. Baseline Characteristics, Imaging Studies, Pathology, and Persistent/Recurrent Disease Patterns and Treatments eReferences eTable1. Demographic Variables and Pre-operative Evaluations eTable2. Pulmonary Neuroendocrine ACTH-Secreting Tumor Clinical Signs/Symptoms eTable3. Wedge/ Segmentectomy Only Patient Characteristics eTable4. Characteristics of Patients With Recurrent Disease eFigure. Approach to the Diagnosis of Cushing’s Syndrome Secondary to Ectopic ACTH Secretion [file jamanetwopen-e2124739-s001.pdf]

Supplemental Online Content

Seastedt KP, Alyateem GA, Pittala K, et al. Characterization of outcomes by surgical management of lung neuroendocrine tumors associated with Cushing syndrome. *JAMA Netw Open*. 2021;4(9):e2124739. doi:10.1001/jamanetworkopen.2021.24739

eMethods. Protocol Approval, Surgical Techniques, Tumor Review, and Analysis

eResults. Baseline Characteristics, Imaging Studies, Pathology, and Persistent/Recurrent Disease Patterns and Treatments

eReferences

eTable1, Demographic Variables and Pre-operative Evaluations

eTable2. Pulmonary Neuroendocrine ACTH-Secreting Tumor Clinical Signs/Symptoms

eTable3. Wedge/ Segmentectomy Only Patient Characteristics

eTable4. Characteristics of Patients With Recurrent Disease

eFigure. Approach to the Diagnosis of Cushing’s Syndrome Secondary to Ectopic ACTH Secretion

This supplemental material has been provided by the authors to give readers additional information about their work.

© 2021 Seastedt KP et al. *JAMA Network Open*.

eMethods

Specific protocol approvals were from the National Institute Child Health and Human Development (NICHD) and National Institute of Diabetes and Digestive and Kidney Diseases (NIDDK) Institutional Review Boards. Ectopic adrenocorticotropic hormone secretion (EAS) was likely if inferior petrosal sinus sampling (IPSS), 8 mg dexamethasone suppression and/or Corticotropin Releasing Hormone (CRH) stimulation tests were consistent with the diagnosis.[1] Such patients underwent additional testing for tumor markers at the endocrinologist's discretion, including urine 5-hydroxyindoleacetic acid (5-HIAA), serum gastrin, plasma and urine catecholamines and metabolites and/ or serum calcitonin. Imaging studies identifying the source of EAS included computed tomography (CT) and/or magnetic resonance imaging (MRI) scans of the neck, chest, abdomen, and pelvis. Some patients also underwent scintigraphy with 6 mCi octreotide, 18 mCi octreotide, ^{18}F-DOPA positron emission tomography (PET)/CT, and/or Gallium-68 Dotatate PET/CT.

Surgical techniques included posterolateral thoracotomy, anterior thoracotomy, and video-assisted thoracoscopic surgery (VATS). The goal of operation was to resect all gross disease for negative margins whenever practicable. Post-operatively, morning serum cortisol, Adrenocorticotropic Hormone (ACTH) and 24-hour urinary cortisol levels were measured. Patients were considered disease-free if the post-operative serum cortisol level was below 5 μg/dL in those who were hypercortisolemic at the time of surgery. Adrenalectomized patients were deemed cured if ACTH decreased to normal adrenalectomized levels and suppressed after 8 mg dexamethasone. Eucortisolemic patients receiving preoperative steroidogenesis inhibitors were considered cured if they had normal 1 mg dexamethasone suppression and a normal cortisol diurnal rhythm. After surgery, patients with persistent endocrinopathy continued follow-up and laboratory/imaging testing to identify EAS.

Tumors were reviewed by pathologists, with most specimens evaluated for ACTH by immunohistochemistry (IHC). Histologic classification as Atypical Carcinoid (AC) was based on World Health Organization (WHO) criteria,[2] with any of these features: increased mitotic rate, pleomorphism, irregular nuclei, or areas of necrosis. Otherwise, tumors were classified as Typical Carcinoid (TC) if none of those features were present. Staging was

© 2021 Seastedt KP et al. *JAMA Network Open*.

updated according to the 8th edition of the American Joint Committee on Cancer (AJCC) system for lung cancer.[3] Tumors without nodal evaluation were categorized as NX. Follow-up information including recurrence, adjuvant treatments, and survival status was identified from the medical record at the last clinical visit or contact and the Social Security Death Index.

For the Kaplan-Meier analysis, patients who completed lung surgery but had ongoing hypercortisolism/ excessive ACTH are defined as persistent and treated as being failures at time zero in the Disease-Free Survival (DFS) curves (i.e., zero duration of being disease-free), while patients who completed lung surgery with no residual hypercortisolism/ excessive ACTH but later redeveloped Cushing's Syndrome (CS) are defined as recurrent and treated as a failure for DFS at the time of recurrence.

© 2021 Seastedt KP et al. *JAMA Network Open*.

eResults

Baseline Characteristics

Prior to presenting, few patients had undergone an endocrine procedural intervention with 11/68 (16%) adrenalectomies and 8/68 (12%) hypophysectomies.

Imaging Studies

Chest CT and MRI localized the target pulmonary lesion(s) in 62/68 (91.2%) and 49/61 (80.3%) of patients, respectively. Of the patients with CS and known pulmonary lesions on imaging, 12/65 (18.5%) of patients also had an abnormal pituitary MRI. Since 2002, PET-based modalities have been used routinely to identify a pulmonary source of CS. Dotatate and ^{18}F-DOPA PET/CT imaging detected lung lesions in 13/20 (65.0%) and 23/37 (62.2%) of patients respectively. Adrenal gland nodules were also noted in 6/60 (10.0%) of patients by abdominal CT imaging. The adrenal lesions were present in the background of bilateral adrenal hyperplasia, and none proved to be ACTH-secreting.

Pathology

The major histologic type was TC (57/68, 83.8%). Tumors were characteristically small with an average diameter of 1.1 cm (range, 0.1-3.5cm). Of patients with at least N1 nodal staging, 22/59 (37.3%) had nodal involvement, and stratified by histopathology, 15/49 (30.6%) of TC had nodal disease while 7/10 (70.0%) of AC had nodal disease. Lymphatic or vascular invasion was found in 11/68 (16.2%) of all resected tumors with 10 of them being AC.

Persistent/Recurrent Disease Patterns and Treatments

Four of seven patients with recurrent tumor underwent adrenalectomy. Six of seven had additional surgical therapy for lung resection with repeated lymph node dissection when indicated, with sub-lobar resection employed in 1/7 of patients. Complete lymph node assessment was performed in 5/7,

© 2021 Seastedt KP et al. *JAMA Network Open*.

with 2/7 having a partial nodal assessment (N1 only). Pathologic nodal disease was found in 4/7, including the lone patient with an AC tumor. Regarding the location of the recurrent tumors, disease occurred locoregionally in 5/7, the contralateral chest in 1/7, or locoregional and distant in 1/7.

Of patients with persistent disease, sub-lobar resection was used in 2/4, with complete nodal dissection performed in 3/4 that yielded negative findings. Persistence of disease after surgery was definitively treated with bilateral adrenalectomy in 1/4, redo-sub-lobar/nodal resection in 1/4, bilateral adrenalectomy plus redo-chest/nodal dissection in 1/4, or medical management in 1/4 until further work up and definitive redo therapy is planned.

Adjuvant therapy was used on a case-by-case basis, including radiation therapy (in a total of 6 patients from our first report[4]), medical therapy to reduce cortisol production (ketoconazole, aminoglutethimide, cabergoline, metyrapone, and/or mitotane), and repeat surgical resections in the chest or bilateral adrenalectomy per the treating physicians.

© 2021 Seastedt KP et al. *JAMA Network Open*.

eReferences

1. Lindsay JR, Nieman LK. Differential diagnosis and imaging in Cushing's syndrome. *Endocrinol Metab Clin North Am* 2005;34:403-421, x.

2. Travis WD, Brambilla E, Nicholson AG, et al. The 2015 World Health Organization Classification of Lung Tumors. *Journal of Thoracic Oncology* 2015;10:1243-1260.

3. Rami-Porta R, Asamura H, Travis WD, et al. Lung cancer - major changes in the American Joint Committee on Cancer eighth edition cancer staging manual. *CA Cancer J Clin* 2017;67:138-155.

4. Pass HI, Doppman JL, Nieman L, et al. Management of the ectopic ACTH syndrome due to thoracic carcinoids. *Ann Thorac Surg* 1990;50:52-57.

© 2021 Seastedt KP et al. *JAMA Network Open*.

eTable1: Demographic Variables and Pre-operative Evaluations

Variable	n (%)
Age (years): median (range)	41 (17-80)
Male	29/68 (42.6)
FEV_1 (%): median (range)	88 (31-116)
Tobacco Use	
Current/History of Use	9/30 (30.0)
Never	21/30 (70.0)
Unknown	38/68 (55.9)
Race	
White	54/66 (81.8)
Black	8/66 (12.1)
Hispanic	4/66 (6.1)
Unknown	2/68 (2.9)
Number of patients treated per decade	
1980s	14/68 (20.6%)
1990s	15/68 (22.1%)
2000s	16/68 (23.5%)
2010s	21/68 (30.9%)
2020s	2/68 (2.9%)
Clinical Presentation	
Cushing’s Syndrome	68/68 (100)
Bronchial symptoms	0/68 (0)
Pre-operative Imaging	
Chest CT	68/68 (100)
Pituitary MRI	65/68 (95.6)
Chest MR	61/68 (89.7)
Abdominal CT	60/68 (88.2)
Abdominal MR	53/68 (77.9)
Octreotide scintigraphy	42/68 (61.8)
^{18}F-DOPA PET/CT	37/68 (54.4)
Gallium-68 Dotatate PET/CT	20/68 (29.4)
Prior Surgical Interventions	
Adrenalectomy	11/68 (16.2)
Hypophysectomy	8/68 (11.8)

FEV_1: Forced Expiratory Volume in 1 second

© 2021 Seastedt KP et al. *JAMA Network Open*.

eTable2: Pulmonary Neuroendocrine ACTH-Secreting Tumor Clinical Signs/Symptoms

Symptom	n (%)
Fatigue/Muscle weakness	58/68 (85.3)
Hypertension	56/68 (82.3)
Hirsutism	28/39 (71.8)
Moon facies	44/68 (64.7)
Truncal adiposity	44/68 (64.7)
Striae (violaceous)	41/68 (60.3)
Psychiatric disorders	38/68 (55.9)
Bruising	37/68 (54.4)
Edema	35/68 (51.5)
Menstrual irregularity	17/39 (43.6)
History of Diabetes Mellitus	26/68 (38.2)
Obesity (Body Mass Index > 30kg/m^2)[a]	14/44 (31.8)
Infections	21/68 (30.9)
Hyperpigmentation	19/68 (27.9)

[a]*Body Mass Index not recorded in 24/68 patients*

© 2021 Seastedt KP et al. *JAMA Network Open*.

eTable3: Wedge/ Segmentectomy Only Patient Characteristics

Patient (year)	Age/Sex	FEV1(%)	Stage	Index Surgery, Extent of nodal dissection	Typical/ Atypical	Persistent/ Recurrence	Time to Recurrence (months)	Follow-up Months	Status at Last Follow-up
1 (1983)	28/F		IA1	Wedge	Typical	Persistent elevation in ACTH and UFC	NA	2	Dead with persistent disease
2 (2003)	55/M		IA1	Wedge, N1+N2	Typical	None	NA	0.5	Alive, tumor free
3 (2006)	68/F		IA1	Wedge, N1+N2	Atypical	None	NA	0.5	Alive, tumor free
4 (2007)	22/F	93	IIIA	Segment, N1+N2	Typical	Recurrent, ipsilateral lung	36	143	Alive with disease
5 (2009)	52/F	82	IA1	Wedge, N1+N2	Typical	None	NA	24	Alive, tumor free
6 (2009)	62/M	94	T1aNX	Wedge	Typical	None	NA	108	Alive, tumor free
7 (2009)	55/F	99	T1aNX	Wedge	Typical	None	NA	21	Alive, tumor free
8 (2010)	60/M		T1bNX	Wedge	Typical	None	NA	0.5	Alive, tumor free
9 (2010)	59/M	90	T1bNX	Wedge	Typical	None	NA	1	Alive, tumor free
10 (2010)	58/F	31	IA2	Wedge, N1+N2	Atypical	None	NA	9	Alive, tumor free
11 (2011)	30/M	62	T1bNX	Wedge	Typical	None	NA	17	Alive, tumor free
12 (2011)	51/F		T1aNX	Wedge	Typical	None	NA	13	Alive, tumor free
13 (2013)	67/F		IA1	Wedge, N1+N2	Typical	None	NA	13	Alive, tumor free
14 (2016)	55/F		T1aNX	Wedge	Typical	None	NA	25	Alive, tumor free

© 2021 Seastedt KP et al. *JAMA Network Open*.

15 (2017)	64/M	104	IIB	Segment, N1+N2	Typical	None	NA	3	Alive, tumor free
16 (2017)	80/F	91	IA2	Wedge, N1+N2	Typical	Persistent, contralateral lung and mediastinum	NA	26	Alive, tumor free
17 (2018)	17/F	116	IIB	Segment, N1+N2	Typical	None	NA	0.1	Alive, tumor free
18 (2018)	22/M	75	IA1	Segment, N1+N2	Typical	None	NA	14	Alive, tumor free
19 (2019)	56/F	76	IA2	Wedge, N1+N2	Atypical	None	NA	0.5	Alive, tumor free

M = Male, F = Female, UFC = urine free cortisol, NX = Nodes not sampled, FEV_1 = Forced Expiratory Volume in 1 second

© 2021 Seastedt KP et al. *JAMA Network Open*.

eTable4: Characteristics of Patients with Recurrent Disease

Patient (year of surgery)	Age/Sex	Time to Surgery (months)	Stage	Index Surgery, Extent of nodal dissection	Typical/ Atypical	Recurrent	Time to Recurrence (months)	Additional Therapy	Follow-up Months	Status at Last Follow-up
1 (1989)	45/F	1	IA1	Lobectomy, N1+N2	Typical	Recurrent, contralateral chest	152	None reported	234	Dead with cancer
2 (1990)	28/M	1	IA1[a]	Lobectomy, N1 only	Typical	Recurrent, ipsilateral hilum	45	Ketoconazole, amoinogluethimide, bilateral adrenalectomy, RT	87	Alive with disease
3 (1990)	30/F	3	IIB[a]	Lobectomy, N1 only	Typical	Recurrent, ipsilateral hilum	114	Thoracotomy and nodal dissection, ketoconazole, cabergoline, hydrocortisone	341	Alive with disease
4 (1990)	33/M	2	IA1	Lobectomy, N1+N2	Typical	Recurrent, ipsilateral lung	111	Thoracotomy, wedge resection, nodal dissection	112	Alive, tumor free
5 (1998)	20/F	1	IIIA	Lobectomy, N1+N2	Atypical	Recurrent, ipsilateral hilum, metastatic to liver	55	Thoracotomy, nodal dissection, bilateral adrenalectomy, hydrocortisone, fludrocortisone	175	Alive with disease
6 (2001)	39/M	1	IIIA	Lobectomy, N1+N2	Typical	Recurrent, ipsilateral mediastinu m	18	Thoracotomy, nodal dissection, mifepristone, repeat VATS nodal dissection, bilateral adrenalectomy	187	Alive with disease
7 (2007)	22/F	1	IIIA	Segment, N1+N2	Typical	Recurrent, ipsilateral lung	36	Thoracotomy, lobectomy, lymph node dissection, bilateral adrenalectomy	143	Alive with disease

M = Male, F = Female, UFC = urine free cortisol, RT = radiotherapy, VATS = Video Assisted Thoracoscopic Surgery

© 2021 Seastedt KP et al. *JAMA Network Open*.

[a] *Partial lymphadenectomy (N1 only), and stage based off of partial nodal staging*

© 2021 Seastedt KP et al. *JAMA Network Open*.

eTable5: Characteristics of Patients with Persistent Disease

Patient (year of surgery)	Age/Sex	Time to Surgery (months)	Stage	Index Surgery, Extent of nodal dissection	Typical/ Atypical	Persistent	Time to Recurrence (months)	Additional Therapy	Follow-up Months	Status at Last Follow-up
1 (1983)	28/F	4	T1aNX	Wedge	Typical	Persistent elevation in ACTH and UFC	NA	Bilateral adrenalectomy	2	Dead with persistent disease
2 (1988)	55/F	7	IA2	Lobectomy, N1+N2	Typical	Persistent elevation in ACTH and UFC	NA	Two redo-thoracotomies, bilateral adrenalectomy	117	Dead with cancer
3 (2017)	80/F	1	IA2	Wedge, N1+N2	Typical	Persistent, contralateral lung and mediastinum	NA	VATS lung wedge resection, nodal dissection	26	Alive, tumor free
4 (2020)	51/M	4	IA2	Lobectomy, N1+N2	Typical	Persistent elevation in ACTH	NA		1	Alive with disease

M = Male, F = Female, UFC = urine free cortisol, RT = radiotherapy, NX = Nodes not sampled, VATS = Video Assisted Thoracoscopic Surgery

© 2021 Seastedt KP et al. *JAMA Network Open*.

eFigure: Approach to the Diagnosis of Cushing's Syndrome Secondary to Ectopic ACTH Secretion

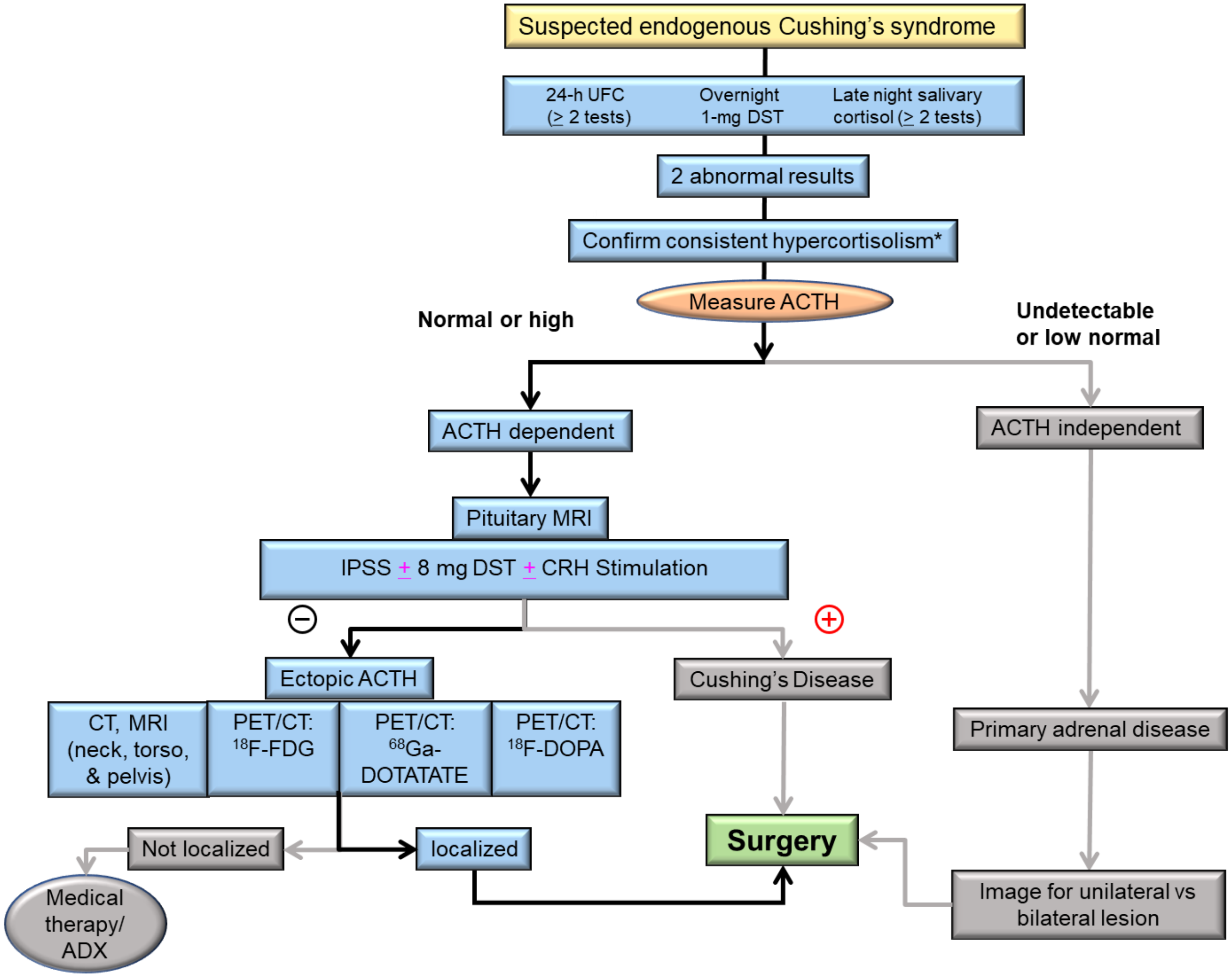

Supplemental Figure 1: Approach to the Diagnosis of Cushing's Syndrome Secondary to Ectopic ACTH Secretion
** UFC and same day bedtime salivary cortisol weekly for 6 weeks*

UFC = Urine Free Cortisol, DST = Dexamethasone Suppression Test, ACTH = Adrenocorticotropic hormone, MRI = Magnetic Resonance Imaging, IPSS = Inferior Petrosal Sinus Sampling, CRH = Corticotropin-Releasing Hormone, CT = Computed Tomography, PET = Positron Emission Topography, ^{18}F-DOPA = 18-Fluorodopa, ^{68}Ga-DOTATATE = Gallium 68-DOTATATE, ^{18}FDG = 18-Fluorodeoxyglucose, ADX = adrenalectomy

© 2021 Seastedt KP et al. *JAMA Network Open*.
